# Intake of ultra-processed foods and accelerated epigenetic aging of colorectal tissues

**DOI:** 10.1093/jncics/pkag092

**Published:** 2026-09-08

**Authors:** Matthew A Devall, Hania M Taha, Nadine Khalil, Xiangqing Sun, Gaizun Hu, Alexa Ramirez, Fatima Z Habib, Samyukta Venkatesh, Mourad W Ali, Joseph Willis, Gregory Cooper, Li Li

**Affiliations:** Department of Family Medicine, University of Virginia, Charlottesville, VA, United States; University of Virginia Comprehensive Cancer Center, University of Virginia, Charlottesville, VA, United States; Department of Family Medicine, University of Virginia, Charlottesville, VA, United States; Department of Family Medicine, University of Virginia, Charlottesville, VA, United States; Department of Family Medicine, University of Virginia, Charlottesville, VA, United States; Department of Family Medicine, University of Virginia, Charlottesville, VA, United States; Department of Family Medicine, University of Virginia, Charlottesville, VA, United States; Department of Family Medicine, University of Virginia, Charlottesville, VA, United States; Department of Family Medicine, University of Virginia, Charlottesville, VA, United States; Department of Family Medicine, University of Virginia, Charlottesville, VA, United States; Department of Pathology, Case Western Reserve University/University Hospitals Cleveland Medical Center, Cleveland, OH, United States; Department of Medicine, Case Western Reserve University/University Hospitals Cleveland Medical Center, Cleveland, OH, United States; Department of Family Medicine, University of Virginia, Charlottesville, VA, United States; University of Virginia Comprehensive Cancer Center, University of Virginia, Charlottesville, VA, United States

## Abstract

**Background:**

Ultra-processed food (UPF) intake is associated with colorectal cancer (CRC) risk and may contribute to rising early-onset CRC incidence, although underlying mechanisms remain unclear.

**Methods:**

We examined associations between UPF intake and accelerated epigenetic aging (AgeAccel) in normal colorectal tissues among 106 participants (73 African American (AA); 33 European American) in the Cleveland Colon Screening and Risk Factors Study (2012-2018). Ultra-processed food intake was assessed using a Food Frequency Questionnaire and classified according to the NOVA system. AgeAccel was derived from DNA methylation assays of colorectal biopsies. Linear mixed-effects regression models adjusted for energy intake, race, and colorectal location assessed associations.

**Results:**

Higher UPF intake was significantly associated with increased AgeAccel in overall colorectum (*P *= 6.87 × 10^−03^; β = .061; SE = 0.028), corresponding to a + 1.41-year difference between the highest and lowest quartiles. Associations were significant in the right colon and among AA participants (*P* < .05).

**Conclusion:**

These findings suggest a potential mechanistic link between UPF intake and CRC risk.

## Introduction 

The NOVA food system classifies ultra-processed foods (UPFs) as products with little whole content that are mainly made of additives and food-derived substances.^1^ In the United States, UPF consumption has increased from ∼10% to more than 60% of total calories.^2^ Numerous studies have aimed to determine the role of this system of foods in complex disease, including colorectal cancer (CRC), where a positive association between UPF and overall CRC risk was found in a recent meta-analysis of 16 cohort studies.^3^

Early-onset CRC (EO-CRC; CRC in individuals <50 years), is rapidly rising in the US,^4^ with incidence increasing by 2.13% annually for individuals 20-49 years of age, and mortality by 1.1% (2005-2023). For individuals younger than 50, CRC is now the leading cause of cancer death and the only cancer to exhibit an increase in mortality between 2005 and 2023.^5^ The causes remain unclear but may include emerging risk factors.^4^ Recent evidence suggests a potential role of UPF intake in early-onset adenomas,^2^ precursors of CRC; however, the underlying molecular mechanisms remain unclear.

Previously, we have shown that accelerated biological aging of the colorectum, as measured by the Horvath epigenetic clock (AgeAccel),^6^ varies by the anatomical sites of the colorectum and by self-reported race, mirroring site-specific risk for CRC/EO-CRC for these racial groups.^7^^,^^8^ AgeAccel is increasingly recognized as a potential biomarker for CRC risk assessment.^9^ In this study, we aim to determine whether UPF intake is associated with normal colorectal tissue AgeAccel.

## Methods 

Individuals included in the present analysis are participants of the Cleveland Colon Screening and Risk Factors Study (2012-2018). Details of the study have been described elsewhere.^7^^,^^8^ Dietary information was collected using a validated semi-quantitative 175-item Food Frequency Questionnaire (FFQ) developed by the Arizona Cancer Center.^10^ Food items were then assigned a binary classifier of 1 (NOVA group 4 (ultra-processed)) or 0 (other food groups) by a registered dietitian after conducting 2 independent full-list reviews of all FFQ items to minimize misclassification and enhance reproducibility. When FFQ items lacked sufficient detail to determine processing level, foods were classified based on the most consumed formulation in the U.S. food supply, consistent with prior NOVA-based analyses of the National Health and Nutrition Examination Survey (NHANES) and cohort studies. The total number of average daily calories (kcal) attributed to each binary group was then calculated for each participant.

Tissue DNA isolation and Illumina EPIC BeadArrays analysis have been previously described.^7^^,^^8^ Raw iDAT files were converted to beta values in minfi^11^ and quality-checked. Epigenetic age acceleration (AgeAccel) was then calculated for each individual sample by regressing DNA methylation age on chronological age using the method described by Horvath.^6^ Linear regression models were then used to test for differences in AgeAccel within colorectal locations. Mixed-effect models were used to identify differences in AgeAccel across colorectal locations. Both analyses assessed the association between the percentage of total calories derived from UPF (herein, %UPF) and AgeAccel as continuous measures. Adjustment covariates were selected based on regression analysis of %UPF and known CRC risk factors. Self-reported race and colorectal location were included in the model because of their previously reported relationship with AgeAccel.^7^^,^^8^

## Results 

Ultra-processed food intake for the 106 analytical individuals is summarized in Table S1, and younger individuals who consumed more calories were significantly more likely to have diets comprising higher UPF (Table S2). A trend was also identified for self-reported race, with African Americans (AAs) consuming an average of approximately 5% more of their total calories from UPF foods than European Americans (EAs).

To determine whether increased %UPF was associated with AgeAccel, we performed a linear mixed-effects regression analysis of all samples combined (*n* = 318; 106 samples per location), adjusting for total caloric intake, self-reported race, and colorectal location. %UPF was significantly associated with AgeAccel overall. In site-specific linear regression models, %UPF was significantly associated with AgeAccel in the right colon (Table 1), with trends observed in the left colon and the rectum.

**Table 1.** Associations of %UPF with AgeAccel in normal colorectal tissues.

Model	Adjusted mean difference per quartile (years)^a^	SE	*t*-value^b^	*P*
**All^c^**	0.061	0.022	2.759	6.87 × 10^−03^
**Right^d^**	0.058	0.029	2.033	.045
**Left^e^**	0.057	0.031	1.811	.058
**Rectum^f^**	0.051	0.027	1.909	.073

Abbreviations: AgeAccel = Age acceleration; UPF = Ultra-processed food.

a Mean difference in AgeAccel between each quartile (1, 2, 3, 4) after accounting for race, total calories, and variation attributed to colorectal location (in full model).

b Positive *t*-values correspond to positive associations between %UPF and AgeAccel.

c This is based on a mixed-effects model of all 318 tissue samples analyzed.

d This is an estimate from linear regression of right colon samples only.

e This is an estimate from linear regression of left colon samples only.

f This is an estimate from linear regression of rectum samples only.

Previously, we have shown that AgeAccel varies by race.^7^^,^^8^ To determine whether UPF-AgeAccel associations differed by race, we stratified the analysis by AA and EA individuals. In AAs, %UPF was significantly associated with overall AgeAccel in mixed-effects models combining all locations (*P *= 2.95 × 10^−04^, *n* = 219), corresponding to a + 2.63-year difference between the first and fourth quartiles. In site-specific linear models, %UPF was significantly associated with AgeAccel at each colorectal location in AAs (*n* = 73 per site; Figure 1). No significant associations were identified in EA individuals.

**Figure 1.**
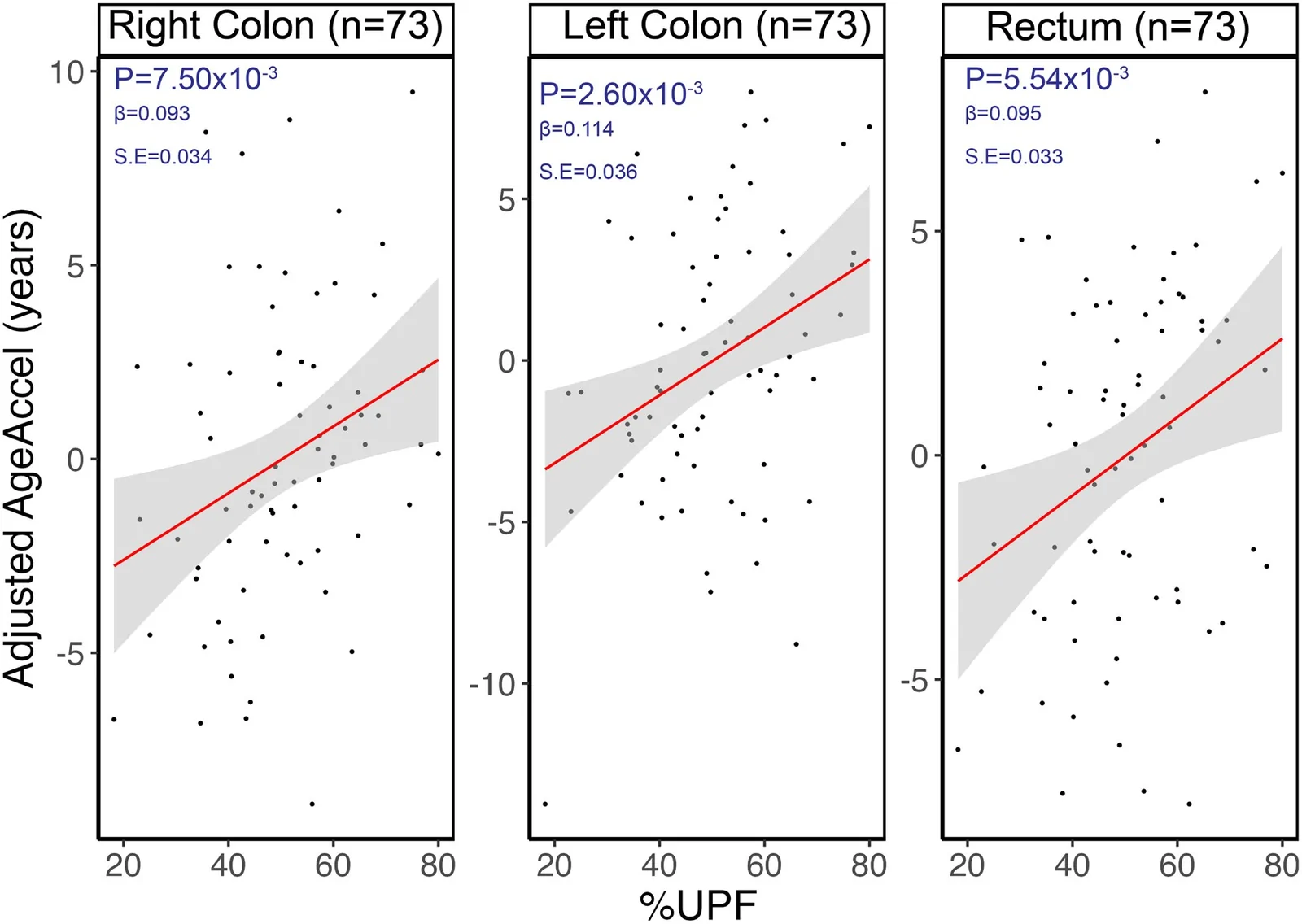
Scatterplot of %UPF and AgeAccel across all 3 colorectal locations of AA individuals (*n* = 219). The red line represents the regression line for each analysis, while the shaded area represents the SE. AgeAccel = Age acceleration; UPF = Ultra-processed food.

## Discussion 

Several epigenetic clocks have been developed to assess accelerated biological aging in relation to complex diseases such as cancer.^12^ Of these, the Horvath AgeAccel clock is one of the most studied, having been trained across multiple tissue types. A recent study based on the NHANES study revealed that this epigenetic clock is the only multi-tissue clock tested to be related to cancer mortality.^12^ In addition, previous research has revealed that individuals with increased AgeAccel were more likely to later develop CRC after a 10-year follow-up.^9^ Together, these findings provide strong support for AgeAccel as a biomarker for cancer-related outcomes. Determining factors that alter the rate of AgeAccel can provide insight into CRC risk etiology and prevention of EO-CRC.

The association between %UPF and Horvath AgeAccel was significant in all 3 colorectal locations of AA individuals. Colorectal cancer arising from different anatomical locations represents different disease identities with distinct molecular features and clinical outcomes.^8^ Better defining factors associated with risk-related markers across the colorectum has the potential to enhance the identification of individuals at greater overall risk. Notably, a previous study of individuals aged between 20 and 45 has revealed that AAs are significantly more likely to be diagnosed with cancer from any colorectal region than EAs.^13^ Whether differences in UPF intake are associated with these observed disparities remains unclear and cannot be determined from the present cross-sectional study. In our study, AA individuals were more likely to report consuming a greater percentage of their total calories from UPF than EAs. It remains unclear whether the non-significant associations observed between %UPF and AgeAccel in EA are due to insufficient sample size or differences in %UPF between the 2 racial groups. For example, only 36% of EA individuals, in contrast to 48% of AA individuals, consumed 50% or more of their total calories from UPF. These findings are similar to those previously reported in larger epidemiological studies, where AAs were significantly more likely to be in the top quartile for UPF consumption than EAs.^14^ However, future larger studies should consider assessment of effect modification to confirm these subgroup-specific differences.

Our study is not without limitations. First, UPF was assessed using a 1-time FFQ only, and information on specific food brands was not obtained. As such, measurement error in UPF assessment is possible. However, the measurement error in UPF is likely to be random, which will tend to attenuate the true association toward the null. Second, because of the cross-sectional nature of our study, we cannot infer causality for the UPF-AgeAcel association. Importantly, a previous randomized controlled trial found that AgeAccel can be significantly reduced following dietary and lifestyle intervention.^15^ Further research is warranted to confirm whether interventions to reduce %UPF will decrease epigenetic aging and CRC risk.

## Conclusion 

In summary, our study provides novel data on the association between UPF consumption and epigenetic age acceleration in normal colorectal tissues, particularly in the right colon and among AA individuals. Prospective studies are needed to determine whether UPF-associated colorectal tissue epigenetic aging contributes to EO-CRC risk.

The authors acknowledge Research Computing at The University of Virginia for providing computational resources and technical support that have contributed to the results reported within this publication.

## Supplementary Material

pkag092_Supplementary_Data

## Data Availability

The data underlying this article are available in Gene Expression Omnibus at https://www.ncbi.nlm.nih.gov/geo/query/acc.cgi, and can be accessed with GSE151732 and GSE216024.
