# Supplementary material for: Intake of ultra-processed foods and accelerated epigenetic aging of colorectal tissues
Source: JNCI Cancer Spectr. 2026 Sep 8;10(5):pkag092. doi: 10.1093/jncics/pkag092 (PMC13637630; doi:10.1093/jncics/pkag092)
Supplement: Supplementary file 1 [file pkag092_supplementary_data.zip › SupplementaryTables.docx]

**Table S1: Sample demographics.**

| ID | Total Calories Consumed | %UPF | Sex | Race | Age | Body Mass Index | Smoking Status |
| --- | --- | --- | --- | --- | --- | --- | --- |
| P3893 | 2866.749 | 56.864 | F | AA | 51 | 21.953 | Current |
| P3897 | 2271.629 | 44.227 | F | AA | 53 | 44.628 | Never |
| P3935 | 1475.042 | 64.691 | M | AA | 54 | 36.023 | Current |
| P3939 | 3057.033 | 50.823 | M | AA | 54 | 38.494 | Current |
| P3952 | 602.942 | 34.672 | M | AA | 51 | 44.816 | Current |
| P3974 | 1059.498 | 45.903 | F | EA | 51 | 26.925 | Never |
| P4004 | 2392.448 | 49.795 | F | AA | 51 | 40.673 | Ever |
| P4015 | 2149.697 | 62.259 | F | AA | 53 | 39.689 | Never |
| P4020 | 1224.189 | 22.955 | M | EA | 60 | 26.387 | Current |
| P4048 | 3829.861 | 40.427 | F | AA | 51 | 36.023 | Current |
| P4052 | 2545.201 | 62.694 | M | EA | 50 | 27.979 | Ever |
| P4055 | 2867.334 | 44.199 | M | AA | 56 | 27.262 | Never |
| P4066 | 4284.420 | 42.660 | M | EA | 61 | 19.734 | Ever |
| P4068 | 1224.939 | 38.118 | M | AA | 72 | 23.989 | Current |
| P4099 | 720.998 | 53.701 | F | AA | 59 | 22.657 | Never |
| P4105 | 2198.920 | 69.369 | F | AA | 49 | 46.345 | Current |
| P4124 | 1154.212 | 49.553 | F | AA | 58 | 30.553 | Never |
| P4125 | 575.415 | 49.724 | F | AA | 56 | 45.725 | Ever |
| P4146 | 706.556 | 41.333 | F | EA | 34 | 33.374 | Current |
| P4158 | 1330.184 | 76.689 | F | AA | 54 | 23.297 | Never |
| P4160 | 1306.785 | 48.405 | F | AA | 54 | 38.059 | Current |
| P4173 | 1438.897 | 51.264 | F | EA | 52 | 23.009 | Ever |
| P4183 | 2200.812 | 44.855 | F | EA | 57 | 37.789 | Never |
| P4193 | 543.214 | 32.675 | M | AA | 60 | 22.107 | Current |
| P4199 | 2531.713 | 46.880 | M | EA | 58 | 22.112 | Ever |
| P4200 | 1824.345 | 37.055 | M | EA | 70 | 26.000 | Never |
| P4202 | 1443.325 | 30.328 | M | AA | 66 | 37.123 | Never |
| P4208 | 1344.235 | 77.012 | M | AA | 59 | 36.483 | Never |
| P4211 | 1871.486 | 36.598 | M | AA | 55 | 28.591 | Current |
| P4223 | 1771.698 | 31.609 | F | EA | 50 | 27.409 | Ever |
| P4239 | 1561.726 | 64.718 | F | AA | 51 | 49.551 | Never |
| P4241 | 2209.699 | 68.817 | M | EA | 39 | 35.186 | Never |
| P4244 | 1729.329 | 47.212 | F | AA | 53 | 37.600 | Current |
| P4270 | 995.089 | 46.297 | F | AA | 65 | 25.127 | Ever |
| P4277 | 2284.486 | 70.738 | M | EA | 67 | 30.132 | Ever |
| P4278 | 1488.111 | 40.574 | M | AA | 54 | 21.698 | Ever |
| P4288 | 2109.741 | 40.142 | M | AA | 56 | 30.273 | Current |
| P4309 | 1255.569 | 44.548 | F | AA | 57 | 44.501 | Current |
| P4310 | 3921.486 | 52.514 | F | AA | 52 | 25.747 | Current |
| P4312 | 1934.431 | 57.276 | F | AA | 53 | 34.158 | Ever |
| P4321 | 2431.173 | 48.978 | M | AA | 57 | 30.108 | Current |
| P4337 | 2718.548 | 60.157 | M | EA | 63 | 35.940 | Never |
| P4346 | 1630.717 | 45.904 | M | AA | 60 | 25.017 | Never |
| P4360 | 772.397 | 60.304 | F | AA | 54 | 30.038 | Current |
| P4364 | 1340.274 | 57.418 | F | EA | 51 | 20.598 | Current |
| P4437 | 1603.761 | 35.704 | M | AA | 51 | 31.092 | Current |
| P4445 | 611.627 | 23.123 | F | AA | 53 | 31.481 | Ever |
| P4459 | 4376.157 | 48.166 | M | AA | 55 | 24.530 | Current |
| P4468 | 1983.041 | 42.624 | M | AA | 79 | 36.491 | Ever |
| P4473 | 2892.494 | 56.912 | M | EA | 67 | 31.532 | Never |
| P4500 | 1629.105 | 48.814 | F | AA | 63 | 34.751 | Ever |
| P4503 | 1436.301 | 39.707 | F | EA | 63 | 26.264 | Current |
| P4524 | 1244.472 | 40.165 | F | AA | 51 | 38.316 | Never |
| P4587 | 1801.972 | 45.989 | F | EA | 49 | 29.758 | Never |
| P4605 | 1285.146 | 13.459 | F | EA | 80 | 34.926 | Ever |
| P4614 | 2407.828 | 52.634 | F | AA | 54 | 21.611 | Never |
| P4688 | 666.437 | 48.456 | F | AA | 63 | 20.526 | Never |
| P4690 | 963.686 | 39.538 | F | AA | 51 | 30.553 | Ever |
| P4694 | 3159.430 | 40.250 | F | AA | 55 | 51.371 | Never |
| P4707 | 2051.441 | 55.964 | F | AA | 60 | 31.248 | Current |
| P4712 | 1183.965 | 18.193 | F | AA | 66 | 30.114 | Ever |
| P4727 | 4313.141 | 58.515 | F | AA | 57 | 28.192 | Never |
| P4759 | 3118.414 | 52.813 | F | EA | 50 | 41.012 | Never |
| P4762 | 1019.945 | 33.870 | F | AA | 56 | 25.340 | Never |
| P4765 | 2609.949 | 56.209 | M | AA | 50 | 32.549 | Never |
| P4774 | 666.313 | 42.866 | F | AA | 51 | 26.958 | Never |
| P4779 | 3285.107 | 41.527 | M | EA | 40 | 20.921 | Never |
| P4784 | 1968.520 | 57.059 | F | AA | 60 | 32.922 | Ever |
| P4796 | 1815.287 | 43.592 | M | EA | 51 | 33.001 | Ever |
| P4797 | 2379.200 | 80.010 | F | AA | 50 | 32.310 | Current |
| P4799 | 1289.590 | 59.893 | F | AA | 50 | 33.643 | Current |
| P4802 | 3071.258 | 52.598 | M | EA | 53 | 28.617 | Never |
| P4804 | 1210.798 | 58.520 | M | EA | 50 | 25.105 | Never |
| P4811 | 1808.009 | 45.712 | F | EA | 56 | 29.405 | Never |
| P4815 | 1336.732 | 22.634 | F | AA | 50 | 31.170 | Ever |
| P4816 | 778.765 | 43.370 | F | AA | 48 | 41.024 | Current |
| P4825 | 4131.927 | 57.042 | F | AA | 50 | 25.848 | Current |
| P4826 | 1740.130 | 53.607 | F | AA | 55 | 32.109 | Ever |
| P4830 | 740.591 | 53.934 | F | AA | 75 | 26.756 | Ever |
| P4846 | 738.571 | 50.227 | F | EA | 66 | 32.449 | Ever |
| P4856 | 1707.260 | 51.682 | M | AA | 55 | 28.123 | Current |
| P4900 | 1154.751 | 59.280 | M | AA | 68 | 23.737 | Ever |
| P4946 | 2321.179 | 34.238 | F | AA | 47 | 32.765 | Never |
| P4952 | 1300.062 | 31.016 | F | EA | 76 | 31.531 | Never |
| P4954 | 3131.384 | 60.113 | M | AA | 60 | 23.292 | Ever |
| P4974 | 2379.261 | 68.582 | F | AA | 65 | 17.218 | Current |
| P5033 | 2583.291 | 61.054 | M | AA | 59 | 32.017 | Ever |
| P5043 | 3568.713 | 57.383 | M | AA | 59 | 36.263 | Never |
| P5076 | 2519.144 | 36.955 | F | EA | 45 | 22.836 | Never |
| P5086 | 2090.246 | 67.776 | F | AA | 53 | 49.090 | Never |
| P5089 | 1543.260 | 63.889 | M | EA | 70 | 38.354 | Ever |
| P5103 | 1254.392 | 30.970 | F | EA | 46 | 20.973 | Never |
| P5132 | 2539.498 | 33.936 | F | EA | 60 | 25.404 | Never |
| P5161 | 1760.864 | 25.128 | F | EA | 70 | 27.464 | Current |
| P5163 | 833.795 | 43.304 | F | EA | 67 | 33.657 | Never |
| P5177 | 2527.192 | 65.294 | M | AA | 50 | 18.631 | Current |
| P5179 | 887.051 | 51.156 | F | AA | 57 | 41.298 | Current |
| P5197 | 2024.873 | 66.057 | F | AA | 54 | 33.895 | Current |
| P5202 | 1420.523 | 35.411 | F | AA | 73 | 43.266 | Ever |
| P5264 | 1522.064 | 46.539 | F | AA | 51 | 33.128 | Ever |
| P5279 | 1520.273 | 63.538 | M | AA | 60 | 18.490 | Current |
| P5283 | 688.003 | 34.653 | F | AA | 73 | 32.120 | Current |
| P5334 | 1028.864 | 40.779 | F | EA | 65 | 20.668 | Never |
| P5339 | 977.425 | 25.047 | F | AA | 53 | 41.504 | Never |
| P5395 | 871.478 | 75.089 | F | AA | 55 | 31.520 | Ever |
| P5458 | 3111.802 | 74.494 | F | AA | 50 | 27.615 | Current |

**Table S2: Summary of regression analysis for CRC related covariates on independent variable (%UPF)**.

| Trait | Mean Difference (years) | Standard Error | t-value | *P* |
| --- | --- | --- | --- | --- |
| Total Calories Consumed | 3.74x10^3^ | 1.40x10^3^ | 2.673 | 8.72x10^3^ |
| Age | -0.321 | 0.160 | -2.008 | 0.047 |
| Race (EA vs AA) | -5.062 | 2.821 | -1.794 | 0.076 |
| Sex (Male vs Female) | 2.664 | 2.753 | 0.968 | 0.335 |
| Smoking (yes/no/never) | 0.915 | 1.565 | 0.584 | 0.560 |
| Body mass index | 0.048 | 0.177 | 0.273 | 0.785 |
